# Supplementary material for: RVG Peptide-Functionalized Favipiravir Nanoparticle Delivery System Facilitates Antiviral Therapy of Neurotropic Virus Infection in a Mouse Model
Source: Int J Mol Sci. 2023 Mar 19;24(6):5851. doi: 10.3390/ijms24065851 (PMC10058582; doi:10.3390/ijms24065851)
Supplement: Supplementary file 1 [file ijms-24-05851-s001.zip › ijms-2211418-supplementary.pdf]

Table S1. List of data of concentrations and peak areas used in the standard curve of T-705.

T-705 concentrations (μg/mL)	Values of peak area
40	878559
30	648929
20	426560
10	213644
5	106126
